# Analysis of the therapeutic interaction provided by a humanoid robot serving stroke survivors as a therapeutic assistant for arm rehabilitation

**DOI:** 10.3389/frobt.2023.1103017

**Published:** 2023-03-06

**Authors:** Thomas Platz, Ann Louise Pedersen, Philipp Deutsch, Alexandru-Nicolae Umlauft, Sebastian Bader

**Affiliations:** ^1^ Neurorehabilitation research group, University Medical Centre, Greifswald, Germany; ^2^ BDH-Klinik Greifswald, Institute for Neurorehabilitation and Evidence-Based Practice, “An-Institut,” University of Greifswald, Greifswald, Germany; ^3^ Department of Computer Science, University of Rostock, Rostock, Germany

**Keywords:** robot, training, arm, stroke, interaction, social, artificial intelligence

## Abstract

**Objective:** To characterize a socially active humanoid robot’s therapeutic interaction as a therapeutic assistant when providing arm rehabilitation (i.e., arm basis training (ABT) for moderate-to-severe arm paresis or arm ability training (AAT) for mild arm paresis) to stroke survivors when using the digital therapeutic system Evidence-Based Robot-Assistant in Neurorehabilitation (E-BRAiN) and to compare it to human therapists’ interaction.

**Methods:** Participants and therapy: Seventeen stroke survivors receiving arm rehabilitation (i.e., ABT [*n* = 9] or AAT [*n* = 8]) using E-BRAiN over a course of nine sessions and twenty-one other stroke survivors receiving arm rehabilitation sessions (i.e., ABT [*n* = 6] or AAT [*n* = 15]) in a conventional 1:1 therapist–patient setting. Analysis of therapeutic interaction: Therapy sessions were videotaped, and all therapeutic interactions (information provision, feedback, and bond-related interaction) were documented offline both in terms of their frequency of occurrence and time used for the respective type of interaction using the instrument THER-I-ACT. Statistical analyses: The therapeutic interaction of the humanoid robot, supervising staff/therapists, and helpers on day 1 is reported as mean across subjects for each type of therapy (i.e., ABT and AAT) as descriptive statistics. Effects of time (day 1 vs. day 9) on the humanoid robot interaction were analyzed by repeated-measures analysis of variance (rmANOVA) together with the between-subject factor type of therapy (ABT vs. AAT). The between-subject effect of the agent (humanoid robot vs. human therapist; day 1) was analyzed together with the factor therapy (ABT vs. AAT) by ANOVA.

**Main results and interpretation**: The overall pattern of the therapeutic interaction by the humanoid robot was comprehensive and varied considerably with the type of therapy (as clinically indicated and intended), largely comparable to human therapists’ interaction, and adapted according to needs for interaction over time. Even substantially long robot-assisted therapy sessions seemed acceptable to stroke survivors and promoted engaged patients’ training behavior.

**Conclusion:** Humanoid robot interaction as implemented in the digital system E-BRAiN matches the human therapeutic interaction and its modification across therapies well and promotes engaged training behavior by patients. These characteristics support its clinical use as a therapeutic assistant and, hence, its application to support specific and intensive restorative training for stroke survivors.

## 1 Introduction

Stroke is the second leading cause of death and a very frequent cause of acquired disability globally with the number of people living with the aftermaths of stroke increasing considerably over the last three decades (GBD, 2016 Stroke Collaborators, 2019).

Neurorehabilitation, the type of medical service providing therapy to promote functional recovery, can reduce stroke-related disability leading to a higher number of people that regain the capacity to care for themselves and, hence, to continue to live on their own (Stroke Units Trialist Collaboration, 2013; Langhorne et al., 2020).

This success is related to the brain’s capacity to recover functionally by reorganizing brain network sub-serving functions (Koch et al., 2021). Recovery of brain function occurs both spontaneously and can be enhanced by specific intensive training of the functions to be restored, that is, by “neural repair therapy” (Joy and Carmichael, 2021).

Indeed, training that addresses impairments (impaired body functions) specifically and with high enough intensity using standardized repetitive training protocols for the targeted functions (Platz, 2004) proved to be superior to conventional therapy even when the same therapeutic time was allocated (Platz et al., 2009). Even though such evidence-based therapy is recommended by international organizations (Platz et al., 2021a), there is a lack of implementation of rehabilitation therapy due to a shortage of skilled staff. This is partially true for high-income countries (HICs), but even more pressing in low- and middle-income countries (LMICs) (Owolabi et al., 2021). As a consequence, there is a need for more specific and intensive “neural repair therapy” that cannot be addressed by the services and human resources available. Also, in future, the demand might further increase secondary to demographic changes (GBD, 2016 Stroke Collaborators, 2019).

Potential solutions for the problem might be an integration of patient-led training or family-led training into the individual rehabilitation process. Unfortunately, the special knowledge necessary to promote functional recovery, the required individual adaptation of specific training schedules, and the necessary motivational requirements for extended periods of training all seem to limit the potential to effectively exploit both patient-led training and family-led training for the rehabilitation of people with neuro-disabilities, for example, after stroke (Tyson et al., 2015; Lindley et al., 2017). High enough training adherence to promote recovery could even not be achieved when patient-led training was assessed as feasible and acceptable by stroke survivors themselves (Horne et al., 2015).

In that situation, and when human resources to provide therapy presumably cannot be expanded to the extent needed to combat stroke-related disability effectively, digital and/or robotic therapeutic systems might be one solution to fill the gap.

The support therapists provide during training-based therapy is complex, that is, providing information including instructions, feedback, and motivating comments, as well as physical guidance and help if necessary. Principally speaking, digital and/or robotic therapeutic systems might serve any of these purposes or even all of them. Their perceived usefulness, for example, the degree to which a person believes that using a particular system would enhance her or his rehabilitation, would rest on any system’s capabilities (Davis, 1989).

Indeed, over the last few decades, mechanical rehabilitation robots, end-effector-based or exoskeleton-type, have been developed that support repetitive training of selective movements for stroke survivors with severe paresis with a need for physical assistance during their exercises (Mehrholz et al., 2018). Such robots offer a high degree of repetitive practice, can track human performance during task execution, and are supported by a substantial body of evidence to be beneficial for restoration of motor function. For each robot, they are, however, limited to only few degrees of freedom (e.g., shoulder and elbow movements only) that they assist to train. Accordingly, their application—while recommended for additional practice (Platz et al., 2021b)—is limited to just few aspects of training and a small subgroup of stroke survivors (for each type of robot). Furthermore, as these systems do not comprehensively guide through therapeutic sessions, there is still the need for close therapeutic supervision during their use in neurorehabilitation and, hence, human resources.

Humanoid robots on the other side have the potential advantage that they can be used as socially interactive robots. Their humanoid appearance might help to build trust in their guidance when their general functionality is well adapted to the service offered by them (Hancock et al., 2011; Jung et al., 2021).

While use cases had been published where socially interactive humanoid robots were designed to provide physical assistance, again the technological affordances for physical help (including safety issues) are complex and, therefore, thus far limit such technology to a small set of tasks to be supported by them. Examples are a robot named RIBA (Robot for Interactive Body Assistance) with human-type arms that is designed to perform heavy physical tasks requiring human contact such as transferring a human from a bed to a wheelchair and back (Mukai et al., 2010), a robotic system for the specific dressing scenario “putting on a shoe” (Jevtić et al., 2019), or a physically interactive humanoid robot application for a human range-of-motion training at the shoulder with skeleton recognition-based motion generation (Miyake et al., 2022).

A further option would be to design a socially active humanoid robot that does not provide physical assistance but acts as a therapeutic assistant without physical contact, hence more like a coach.

A strength of such a dedicated system would be that it could be conceptualized to comprehensively guide through therapeutic sessions, considerably reducing the need for close therapeutic supervision during their use in neurorehabilitation. Also, such a humanoid robot-based digital therapeutic system could be designed, developed, and consequently used as a platform for a wide variety of types of neurorehabilitation training therapy. For that purpose, such systems should both have artificial intelligence (AI) embedded that guarantees the individualized application of the professional knowledge necessary during training sessions and sufficiently support motivational factors to ensure prolonged engaged training even among people with brain damage.

Technology that provides one aspect only (e.g., digital health applications with training schedules) may fall short of the needs of people with neuro-disabilities being candidates for restorative training.

Socially interactive robots might provide a technology base to address the interpersonal aspects of training more sufficiently. Indeed, as human beings, we are inclined to accept a humanoid robot as a kind of social partner (Darling et al., 2015). Also, interviews with stroke survivors who underwent a long-term rehabilitation process, assisted by either a socially interactive humanoid robot or a computer interface, support the notion that socially interactive humanoid robots augment rehabilitative therapies beyond a standard computer (Koren et al., 2022).

The digital therapy system Evidence-Based Robot-Assistant in Neurorehabilitation (E-BRAiN) (https://www.ebrain-science.de/en/home/) that was used in this research project allows a humanoid robot to lead stroke survivors receiving rehabilitation treatment through therapeutic sessions, to give instructions for carefully selected training exercises, provide feedback, and support their motivation. In this context, the robot’s task is not to take therapeutic decisions, but to autonomously continue a repetitive training schedule once it has been decided on, which was individually adapted and introduced to a stroke survivor by a human therapist (Forbrig et al., 2022).

The digital therapy system E-BRAiN was specifically designed to be used as a socially interactive humanoid robot as technology, to establish AI that provides (A) professional therapeutic training knowledge for both arm rehabilitation and neglect therapy based on types of therapy with evidence to support their effectiveness for recovery post stroke, (B) to lead through (daily) therapy sessions in an autonomous way with all communication and therapeutic interaction necessary, and (C) to individualize all activities based on individual data (e.g., clinical characteristics, results of assessment, therapeutic goal, and progress made during training).

The system further supports referring expressions in a real-time text-generation system so that generated texts can be adapted to the user in the best possible way (Felske et al., 2022).

In consequence, the therapeutic interaction is complex including provision of information (related to individual rehabilitation goals, training specifications, and training instructions), feedback (in the form of knowledge of result or performance, with or without additional social stimuli), and bond-related interactions (showing interest in the person treated).

Equipped in this way, the digital therapy system E-BRAiN is now used to treat stroke survivors.

The aim of this research was to characterize the humanoid robot’s therapeutic interaction when providing arm rehabilitation (i.e., arm basis training (ABT) for moderate-to-severe arm paresis or arm ability training (AAT) for mild arm paresis) to a group of stroke survivors and its change over time (from the first to the last session with the robot) when using the digital therapeutic system E-BRAiN and to compare the humanoid robot’s therapeutic interaction to human therapists’ interaction.

The sample of stroke survivors using the system E-BRAiN for arm rehabilitation are participants of a clinical trial to test the system’s acceptability, safety, and clinical benefit (University Medicine Greifswald, 2021). Observations made with the control sample of stroke survivors result from the provision of the same therapies in the same context, but in the conventional 1:1 human therapist–patient setting.

## 2 Methods

### 2.1 Technical characterization of the E-BRAiN system

#### 2.1.1 Technical setup (hardware)

**FIGURE 1:**
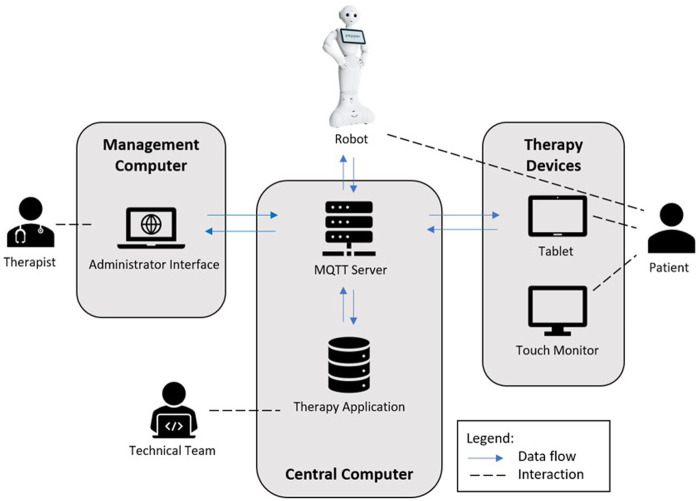
In this figure, the technical setup and roles of the patients, therapists, and technical team (programmers) are depicted. All devices are connected *via* Wi-Fi in the same network. The main program files of the system are hosted on a central computer running on Cent OS (Linux operating system; https://www.linux.org). The software of the therapy application consisting of the python dialog scripts, database, therapy management interface, and communication is deployed on this computer. During a therapy session, all device interactions are transmitted *via* the OASIS standard messaging protocol for the IoT MQTT (https://mqtt.org/) and processed on the computer. The main focus is to offer therapy sessions for the patient. To organize a therapy and its sessions, a therapist creates a patient entry in the system database *via* the therapy administration interface and configures the therapy sessions. The interface can be accessed on any generic computer inside the system network. The humanoid robot Pepper (https://www.unitedrobotics.group/products-services/hardware/), the Android OS tablet, and the touch monitor are running apps developed for the therapies. The robot provides the verbal dialog to patients while the other devices are used in parallel to display images, videos and subtitles (e.g., for instruction purposes), diagrams (e.g., for knowledge of result feedback), or plain text (e.g., “do you need a break?“) and to support the robot program with patients’ entries (e.g., “ready to continue”). A system programmer of the technical team has access to the system to check for errors and possibly fix errors on the application.

The robot system consists of multiple devices with a central architecture, as presented in Figure 1.

In this figure, the technical setup and roles of the patients, therapists, and technical team (programmers) are depicted (Forbrig et al., 2021). All devices are connected *via* Wi-Fi in the same network. The main program files of the system are hosted on a central computer running on Cent OS (Linux operating system; https://www.linux.org). The software of the therapy application consisting of python dialog scripts, database, therapy management interface, and communication is deployed on this computer. During a therapy session, all device interactions are transmitted *via* the OASIS standard messaging protocol for the Internet of Things (IoT) MQTT (https://mqtt.org/) and processed on the computer. The main focus is to offer therapy sessions for the patient. To organize a therapy and its sessions, a therapist creates a patient entry in the system database *via* the therapy administration interface and configures the therapy sessions. The interface can be accessed on any generic computer inside the system network. The humanoid robot Pepper (https://www.unitedrobotics.group/products-services/hardware/), the Android OS tablet, and the touch monitor are running apps developed for the therapies. The robot provides the verbal dialog to patients while the other devices are used in parallel to display images, videos, and subtitles (e.g., for instruction purposes), diagrams (e.g., for knowledge of result feedback), or plain text (e.g., “do you need a break?”) and to support the robot program with patients’ entries (e.g., “ready to continue”). The touch monitor with a 27-inch screen is used for the neuro-visual therapy of the project (not used in this study population). A system programmer of the technical team has access to the system to check for errors and possibly fix errors on the application.

#### 2.1.2 Robot control algorithm

For a flexible and precise determination, of what content and robot feedback is provided at a certain point of a therapy, the therapy interaction is designed around the concept of a “finite-state machine” (Bundea et al., 2021). The robot operates in these therapy “states,” a small part of the therapy program script. The robot starts at the “start” therapy state and proceeds to the next state either after a pre-defined time or a patient confirmation until the final state “saying goodbye” has been reached.

Therapy states are linked with media content and robot actions to be executed at the point of time, when a state is called. When the therapy dialog script transits to another therapy state, a message will be sent to all connected devices, which involves the robot and either the tablet or monitor. The devices will then interpret the message and execute possible commands such as displaying videos or providing speech feedback.

This design of therapy states allows for a flexible robot control, whereby being able to pause at any given therapy state and (re-)entering any other therapy state are the most important features. This control pattern and therapy design also helped to ensure a patient sees exactly the pre-defined contents in the correct order.

#### 2.1.3 Content of social interaction

Stroke survivors need intensive specific training schedules, frequently for a prolonged period. For the realization of such training schedules, patients frequently have to be provided with close supervision and professional guidance based on therapeutic interaction guaranteeing information provision and specific individualized feedback as well as work alliance and motivation supporting personal contact.

With the E-BRAiN system, the humanoid robot’s social interaction is set up to fulfill all of these requirements with standards for each type of training implemented (e.g., the AAT and the ABT) and dialog structures for complete training sessions starting with a personalized “welcome” to closing the therapeutic session.

Specifically, the humanoid robot welcomes the patient individually, explains (A) the therapeutic goal, (B) the prescribed therapy and how it works, and (C) individual training tasks, (D) provides instructions audiovisually (using photos and videos), (E) gives feedback according to the type of therapy and any progress, and (F) asks and provides breaks as needed.

The therapeutic interaction is individualized, based on knowledge about the patient from the medical chart, assessments made before training, and therapeutic progress during training sessions.

### 2.2 Participants

Participants for this study could be stroke survivors who participated in the clinical trial E-BRAiN (https://clinicaltrials.gov/ct2/show/NCT05152433) and completed the 2-week course of humanoid robot-led therapy. Eligibility criteria for the E-BRAiN trial are as follows: age ≥ 18 years, history of stroke (ischemic stroke, non-traumatic intracerebral hemorrhage, and subarachnoidal hemorrhage), either stroke-related upper extremity paresis or visual neglect, not pregnant or breastfeeding, not living in custody, and providing informed consent.

Data of the first 17 participants of the trial receiving arm rehabilitation as either ABT for moderate-to-severe arm paresis or AAT for mild arm paresis were planned to be used for this study.

The sample of control subjects receiving therapy in the conventional 1:1 human therapist–patient setting was a convenience sample of 21 participants of age ≥ 18 years, with a history of stroke (ischemic stroke, non-traumatic intracerebral hemorrhage, and subarachnoidal hemorrhage), and stroke-related incomplete upper extremity paresis interested in a 1-week course of complimentary intensive daily arm rehabilitation (ABT or AAT), and providing informed consent.

The research was approved by the institution review board (Ethikkommission der Universitätsmedizin Greifswald; date of approval: 10.05.2021).

### 2.3 Participant characteristics

For all participants, the following characteristics were documented at study entry: age, gender, types of stroke etiology (i.e., ischemic stroke, non-traumatic intracerebral hemorrhage, or subarachnoidal hemorrhage), time post-stroke (in weeks), and degree of neuro-impairment (National Institute of Health Stroke Scale (NIHSS)) (Brott et al., 1989) and neuro-disability (Barthel Index) (Mahoney and Barthel, 1965), as well as emotional distress (Hospital Anxiety and Depression Scale (HADS)) (Snaith, 2003), in addition to arm motor function (i.e., Box and Block Test (BBT) for participants with mild arm paresis or Fugl–Meyer Arm Motor score (FM Arm) for participants with moderate-to-severe arm paresis) (Fugl-Meyer et al., 1975; Platz et al., 2005).

### 2.4 Therapies applied

The AAT is based on eight training tasks addressing different sensorimotor abilities such as aiming, steadiness, speed of finger movements, and finger and gross manual dexterity. With the AAT, stroke survivors train their sensorimotor efficiency by repetitively executing each task at their individual performance limit in (four) blocks (each) lasting approximately 1 minute and constantly trying to improve their speed of execution while keeping the required level of precision. During the AAT, both the human therapist and humanoid robot provide information including instructions and feedback. The feedback is given as intermittent summary knowledge of result (KR) both with the time needed for each block of execution (per task) showing within-session progress for each task separately and the average time across (four) blocks for each day and task compared to the corresponding measure from the previous days of training indicating the learning process across days.

The ABT trains selective movement capacity for individual joints of the shoulder, elbow, forearm, wrist, and fingers by repetitive movement attempts across the full range of passive movements in the various directions possible in these joints. Each day, all movements are addressed individually and repetitively in a consecutive sequential way. Since patients receiving the ABT have moderate-to-severe arm paresis and cannot perform these movements, or only to a limited extent, or only without weight-bearing affordances, they are physically assisted as needed during the training (Platz, 2004; Platz et al., 2009). Each movement (depending on an individual’s capacity) may be performed without the need for weight bearing of the limb (weight bearing is taken over by a therapist) or alternatively against gravity or with gravity influence (for subjects able to control weight bearing of their limb segments). All individual movements are prompted by a therapist, then attempted by the trainee, and might be completed to the extent individually needed by a therapist coupled with the patient’s intention to move. Feedback is given by therapists as knowledge of performance (KP), that is, the degree as to which selective innervation and movement could be executed (in the intended joint, e.g., shoulder, elbow, forearm, wrist, or fingers) by a patient during the prompted attempt to perform the specific movement requested.

The following aspects of training implementation are specific for the situation with humanoid robot-led training: participants receive a first introductory session with a human therapist where they learn to know and how to perform the standardized training (AAT or ABT), its tasks, focus of motor control, and sequence of events. During this introductory session, the human therapist also notes and decides on individualizations indicated for either the AAT or the ABT that will then be used as prescription for the digital therapy system E-BRAiN.

For this research, the therapeutic training was led by the humanoid robot (“robot”) during the consecutive nine sessions providing therapeutic interaction as implemented in the digital system based on both training standards and individualization algorithms. For safety reasons and to step in if needed, all humanoid robot-led sessions were accompanied by the supervising staff (“therapist”). Since the robot cannot provide physical assistance, but serves as a social agent only (providing therapeutic interaction), participants receiving the ABT were given physical assistance as needed by a “helper.” The helper was not a trained therapist, but similarly used the instructions provided by the robot.

**FIGURE 2:**
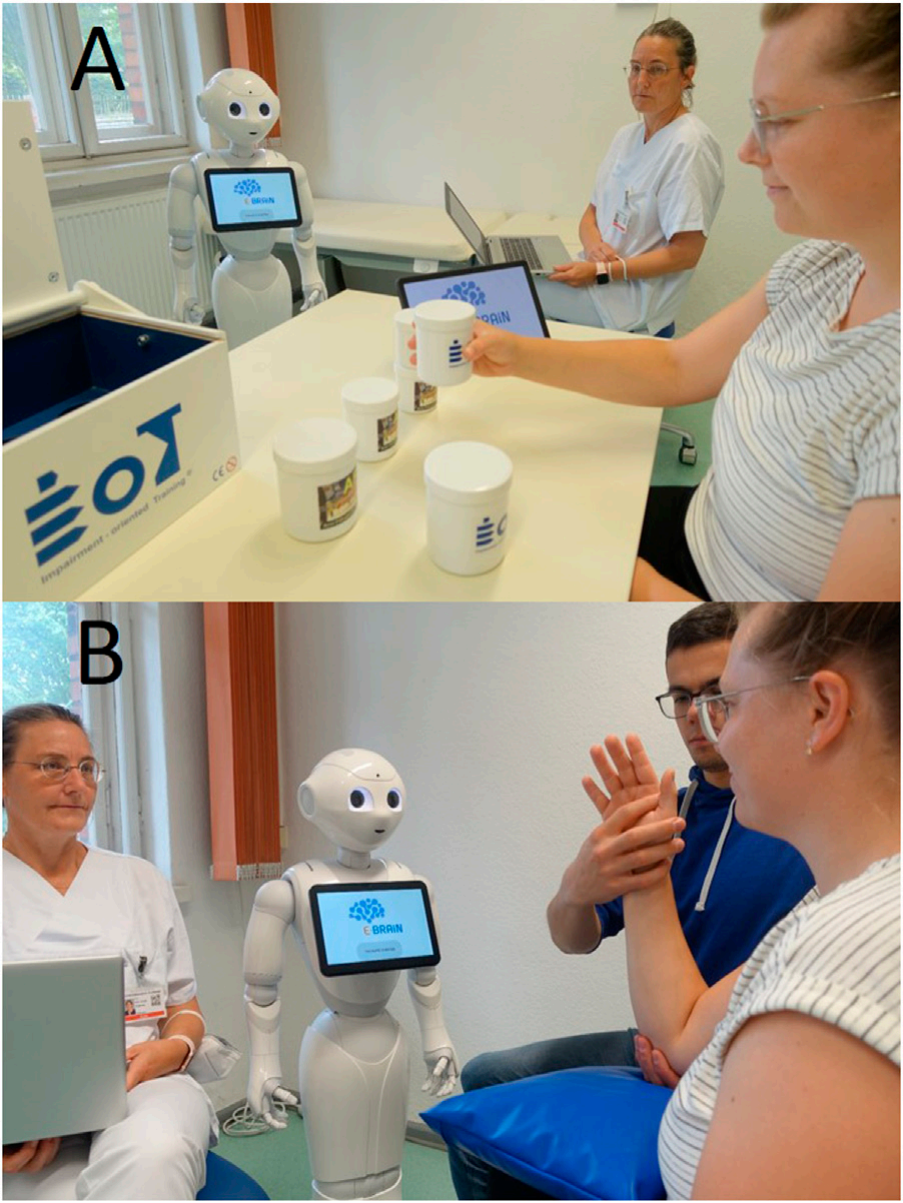
The therapeutic scenarios for the digital therapeutic system E-BRAiN using a humanoid robot to provide therapeutic interaction during arm rehabilitation sessions [i.e., AAT for mild arm paresis **(A)** and ABT for moderate-to-severe arm paresis **(B)**] for stroke survivors. It should be noted in the scenario for the AAT (A), the patient is able to train the (mildly) affected right arm self-sufficiently; here, the robot provides all therapeutic interactions (information provision, feedback, and bond-related interaction) while the patient is led through a sequence of training tasks; the supervising staff in the background is only monitoring the situation and ready to step in in case the system designed to run autonomously showed an error or a patient’s need could not be met by the system. The situation for the ABT is similar with regard to the role of the humanoid robot and supervising staff; here, however, a helper is integrated as a third active agent (in addition to the patient and humanoid robot), a person not qualified as a therapist, who is also guided by the humanoid robot and provides physical assistance as needed for the training of a severely paretic arm.

The two scenarios for humanoid robot-led therapy are depicted in Figure 2 (A, AAT; B, ABT).

### 2.5 Therapeutic context

Participants received their therapy as either outpatients at the University Medical Centre Greifswald or inpatients (sub-acute rehabilitation) in the BDH-Klinik Greifswald, in rooms with typical equipment for rehabilitation therapy, and the timing of their daily study-related therapy adapted to their individual schedules.

### 2.6 Analysis of the therapeutic interaction

Therapy sessions were videotaped, and all therapeutic interactions (information provision, feedback, and bond-related interaction) were documented offline both in terms of their frequency of occurrence and time used for the respective type of interaction during therapy sessions with standardized criteria using the instrument THER-I-ACT.

Using the instrument THER-I-ACT, various types of therapy-related communication interactions performed by therapists can be assessed with a high inter-rater reliability (Platz et al., 2021a). In addition, the thematic fields and categories of therapeutic interaction as defined by the instrument comprehensively cover the types of interaction that occur in therapeutic sessions. This is also true for situations where therapy is led by a humanoid robot (Platz et al., 2023).

For both the robot- and human therapist-led therapy, the therapeutic interaction during the first session with the respective agent (i.e., robot or human therapist) was analyzed; for the robot-led therapy, the last (9^th^) session of daily training with the robot was analyzed in addition.

For the sessions with a humanoid robot, any additional therapeutic interaction spontaneously provided by the supervising staff or human helper needed to provide physical assistance (ABT only) was also documented.

### 2.7 Statistical analyses

Baseline characteristics and therapy assignment (i.e., ABT or AAT) are presented using descriptive statistics, that is, mean and standard deviation (s.d.), or count and relative frequency as indicated, for both the group receiving therapy using the digital therapy system E-BRAiN and the group receiving therapy in the conventional setting with a human therapist, respectively. Statistical analyses for baseline differences between these groups were performed using two-way chi-square tests or two-sample (independent group) t-tests as indicated; for t-tests, the equality of variances for the two groups had been tested with F tests; t-tests for equal or unequal variances had been used accordingly.

Humanoid robot, supervising staff, and helper interaction on day 1 is reported as mean across subjects for each type of therapy (i.e., ABT and AAT, resp.) as descriptive statistics. Effects of time (day 1 vs. day 9) on the humanoid robot interaction were analyzed by repeated-measures analysis of variance together with the between-subject factor type of therapy (ABT vs. AAT). The between-subject effect of the agent (humanoid robot vs. human therapist) was analyzed together with the factor therapy (ABT vs. AAT) by analysis of variance (ANOVA).

### 2.8 Sample size calculation

The statistical corroboration of bigger intergroup differences (effect size f = 0.5) with a pre-defined alpha error probability of 0.05 and power (1 – beta error probability) of 0.80 required a sample of 34 participants; to corroborate statistically (alpha error probability 0.05, power 0.80) at least substantial changes of humanoid robot behavior over time (effect size f 0.40), a sample size of 15 participants in the subgroup with robot-led therapy was necessary (Faul et al., 2013).

## 3 Results

### 3.1 Participants

Data of 17 stroke survivors receiving arm rehabilitation sessions (i.e., ABT [*n* = 9] or AAT [*n* = 8]) using a humanoid robot as a therapeutic agent over a course of nine sessions and 21 other stroke survivors receiving arm rehabilitation sessions (i.e., ABT [*n* = 6] or AAT [*n* = 15]) in a conventional 1:1 therapist–patient setting were used for the purpose of this study.

The study population (and both sub-groups) showed a considerable age distribution, both genders, different types of stroke etiology, a considerable variability of time post-stroke ranging from a few weeks to years, and mild-to-moderate neuro-impairment (NIHSS) (Brott et al., 1989) and neuro-disability (Barthel Index) (Mahoney and Barthel, 1965), as well as emotional distress (HADS) (Snaith, 2003). Similarly, within the sub-groups with either mild or moderate-to-severe arm paresis, the degree of arm and hand motor (dys) function varied considerably (comparing BBT and FM Arm scores, respectively) (Fugl-Meyer et al., 1975; Mathiowetz et al., 1985; Platz et al., 2005).

Accordingly, the sample might well present the variation typically seen in stroke survivors seeking neurorehabilitation services and, hence, challenges for the therapeutic system to address and adapt therapeutic interaction to diversified individual needs during rehabilitation therapy sessions.

Differences noted between the two sub-groups were a higher percentage of female participants in the subgroup receiving robot-led therapy and, on average, more pronounced neuro-disability (lower BI scores) and more severe motor impairment (FM Arm, group with moderate-to-severe arm paresis) in the group receiving robot-led therapy (comparing Table 1).

**TABLE 1:** Study population characteristics (*n* = 38).

	Robot therapy (*n* = 17)			Human therapist (*n* = 21)			P
	Mean/sd, n (%)	Min–max, n (%)	n	Mean/sd, n (%)	Min–max, n (%)	N	
**Age** (mean/sd, min–max)	62.4/14.3	36–81		65.0/9.2	49–80		0.5110 (t)
**Sex** (female, male) (n (%))	11 (65%)	6 (35%)		6 (29%)	15 (71%)		0.0259 (chi)
**Stroke type** (ischemic, ICH) (n (%))	14 (82%)	3 (18%)		18 (86%)	3 (14%)		0.7775 (chi)
**Affected brain** (left, right) (n (%))	6 (35%)	11 (65%)		11 (52%)	10 (48%)		0.2922 (chi)
**Time post-stroke (weeks)** (mean/sd, min–max)	86/115	3–367		212/315	8–1158		0.1006 (t)
**NIHSS** (0–42)(mean/sd, min–max)	4.6/2.1	1–9		6.4/4.8	2–18		0.1454 (t)
**Barthel Index** (0–100)(mean/sd, min–max)	79/18	35–100		92/10	70–100		0.0132 (t)
**HADS** (0–42)(mean/sd, min–max)	12.3/5.9	6–25	16^1^	10.6/7.0	2–27	20^1^	0.4407 (t)
**FM Arm^a^** (0–66) (mean/sd, min–max; n)	20.6/6.7	12–30	9	31.3/9.8	20–49	6	0.0244 (t)
**BBT^b^** (blocks/minute) (mean/sd, min–max; n)	32.5/11.6	18–44	8	38.3/11.3	17–58	15	0.2622 (t)
**Type of therapy** (ABT^a^, AAT^b^)	8	9		6	15		0.1265 (chi)

AAT , arm ability training; ABT, arm basis training; BBT, Box and Block Test; chi, *p*-value for the two-way chi-square test; FM Arm, Fugl–Meyer Arm Motor score; HADS, Hospital Anxiety and Depression Scale; ICH , intracerbral hemorrhage; NIHSS, National Institute of Health Stroke Scale; t, *p*-value for the two-sample (independent group) t-test.

Superscript letters (AAT^a^ and ABT^b^) indicate the different types of therapy and how they relate to both the treated syndromes and the tests used for baseline assessment, respectively.

^a^ One participant in each group did not want to disclose personal emotional information.

### 3.2 Therapeutic interaction

#### 3.2.1 Pattern of the therapeutic interaction by the humanoid robot, supervising staff, and a helper

The pattern of therapeutic interaction as provided by the humanoid robot included episodes of provision of information, feedback, and bond-related interaction (compare Table 2). The therapeutic interaction varied markedly with the type of training (ABT or AAT) as warranted clinically and intended (comparing, also, Tables 3, 4 including statistical analyses for factor “therapy”).

**TABLE 2:** Ther-I-Act observations: Therapeutic interaction by the humanoid robot, supervising therapist, and helper (day 1) (*n* = 17).

**Themes and individual aspects**	**Humanoid robot** (mean)				**Supervising therapist** (mean)				**Helper (ABT only)** (mean)	
	**ABT** (*n* = 9)		**AAT** (*n* = 8)		**ABT** (*n* = 9)		**AAT** (*n* = 8)		**ABT** (*n* = 9)	
	**Fr**	**Ti**	**Fr**	**Ti**	**Fr**	**Ti**	**Fr**	**Ti**	**Fr**	**Ti**
1. Provision of information										
a. Treatment goal	4.6	227	2.0	140	0.1	< 1	0	0	0	0
b. Training specifications	0	0	1.0	290	0	0	0	0	0	0
c. Instructions	388	1859	145	1352	2.1	8	30	131	42.8	93
2. Feedback										
a. Knowledge of performance (unless corrective),	0	0	0	0	0.1	< 1	0.6	< 1	4.4	4
b. KP and positive social stimuli	0	0	0	0	0.4	< 1	0	0	1.2	1
c. KP and negative social stimuli	0	0	0	0	0	0	0	0	0	0
d. Corrective KP (cKP)	0	0	0	0	0	0	0	0	0	0
e. cKP and positive social stimuli	0	0	0	0	0	0	0	0	0	0
f. cKP and negative social stimuli	0	0	0	0	0	0	0	0	0	0
g. Knowledge of result	0	0	35.9	313	0	0	0.9	1	0	0
h. KR and positive social stimuli	0	0	4.3	34	0	0	0.1	< 1	0	0
i. KR and negative social stimuli	0	0	0	0	0	0	0	0	0	0
3. Motivational interactions										
a. Other than KP or KR	0.9	7	2	13	0	0	0.5	2	0.3	< 1
4. Bond										
a. Showing interest in person	16.7	118	44.5	289	1.2	19	4	30	12.9	29
b. Personal aspects (therapist)	0	0	0	0	0	0	0	0	0	0
c. Responsivity	0	0	0	0	0.1	< 1	5.4	16	0.8	1
d. Conflict solving	0	0	0	0	0.1	4	0	0	0.1	< 1
5. Other types of interaction	0	0	0	0	0.3	1	0.5	2	0.4	2
6. Presence (concentration) and engagement (treating person) (0–10)	5		5		8.9		9.3		9.9	
7. Focussed attention and engagement (patient) (0–10)	8.3		8.8							
Length of the therapeutic session (minutes)	77		107							

ABT, arm basis training; AAT, arm ability training; Fr, frequency of occurrence of the therapeutic interaction within the session (count) rounded to one decimal; Ti, time used for the therapeutic interaction within the session (in seconds) rounded to full seconds; KP, knowledge of performance; KR, knowledge of result.

**TABLE 3:** Ther-I-Act observations: Variation of the therapeutic interaction by the humanoid robot with therapy and over time (*n* = 17).

**Themes and individual aspects**	**Day 1 of therapy (mean)**				**Day 9 of therapy (mean)**				**Effect (*P* [F-test])**			
	**ABT** (*n* = 9)		**AAT** (*n* = 8)		**ABT** (*n* = 9)		**AAT** (*n* = 8)		**Therapy**		**Day**	
	**Fr**	**Ti**	**Fr**	**Ti**	**Fr**	**Ti**	**Fr**	**Ti**	**Fr**	**Ti**	**Fr**	**Ti**
1. Provision of information												
a. Treatment goal	4.6	227	2.0	140	2.6	75	2.0	72	**<.0001**	**0.0041**	**<.0001**	**<.0001**
b. Training specifications	0	0	1.0	290	0.8	17	0.8	27	**0.0005**	**<.0001**	**0.0294**	**<.0001**
c. Instructions	388	1859	145	1352	430	1962	156	805	**0.0001**	**0.0007**	**0.0138**	**<.0001**
2. Feedback												
a. Knowledge of performance (unless corrective)	0	0	0	0	0	0	0	0	n.a.	n.a.	n.a.	n.a.
b. KP and positive social stimuli	0	0	0	0	0	0	0	0	n.a.	n.a.	n.a.	n.a.
c. KP and negative social stimuli	0	0	0	0	0	0	0	0	n.a.	n.a.	n.a.	n.a.
d. Corrective KP (cKP)	0	0	0	0	0	0	0	0	n.a.	n.a.	n.a.	n.a.
e. cKP and positive social stimuli	0	0	0	0	0	0	0	0	n.a.	n.a.	n.a.	n.a.
f. cKP and negative social stimuli	0	0	0	0	0	0	0	0	n.a.	n.a.	n.a.	n.a.
g. Knowledge of result	0	0	35.9	313	0	0	34.6	314	**<.0001**	**<.0001**	0.3726	0.9598
h. KR and positive social stimuli	0	0	4.3	34	0	0	5.3	38	**<.0001**	**<.0001**	0.3811	0.6499
i. KR and negative social stimuli	0	0	0	0	0	0	0	0	n.a.	n.a.	n.a.	n.a.
3. Motivational interactions												
a. Other than KP or KR	0.9	7	2	13	0.4	6	1.6	14	**<.0001**	**0.0045**	**0.0184**	0.9588
4. Bond												
a. Showing interest in person	16.7	118	44.5	289	17.3	115	50.9	304	**<.0001**	**<.0001**	**<.0001**	0.4360
b. Personal aspects (therapist)	0	0	0	0	0	0	0	0	n.a.	n.a.	n.a.	n.a.
c. Responsivity	0	0	0	0	0	0	0	0	n.a.	n.a.	n.a.	n.a.
d. Conflict solving	0	0	0	0	0	0	0	0	n.a.	n.a.	n.a.	n.a.
5. Other types of interaction	0	0	0	0	0	0	0	0	n.a.	n.a.	n.a.	n.a.
6. Presence (concentration) and engagement (treating person) (0–10)	5.0		5.0		5.0		5.0		n.a.		n.a.	
7. Focussed attention and engagement (patient) (0–10)	8.3		8.8		8.4		8.9		0.5283		0.3474	
Length of the therapeutic session (minutes)	77		107		74		68		0.0853		**<.0001**	

ABT, arm basis training; AAT, arm ability training; Fr, frequency of occurrence of the therapeutic interaction within the session (count) rounded to one decimal; Ti, time used for the therapeutic interaction within the session (in seconds) rounded to full seconds; KP, knowledge of performance; KR, knowledge of result; *p*-values correspond to F statistics based on type III sums of squares of ANOVA. Bold values denote *p*-values < .05.

**TABLE 4:** Ther-I-Act observations: Variation of the therapeutic interaction by agent and type of therapy (day 1 of therapy) (*n* = 38).

**Themes and individual aspects**	**Human interaction (mean)**				**Robot interaction (mean)**				**Effect (*P* [F-test])**			
	**ABT (*n* = 6)**		**AAT (*n* = 15)**		**ABT (*n* = 9)**		**AAT (*n* = 8)**		**Agent**		**Therapy**	
	**Fr**	**Ti**	**Fr**	**Ti**	**Fr**	**Ti**	**Fr**	**Ti**	**Fr**	**Ti**	**Fr**	**Ti**
1. Provision of information												
a. Treatment goal	1.5	225	1.3	124	4.6	227	2.0	140	**<.0001**	0.6365	**<.0001**	**0.0002**
b. Training specifications	1.2	80	1.4	75	0	0	1.0	290	**0.0032**	**0.0029**	**0.0112**	**<.0001**
c. Instructions	567	1970	159	1055	388	1859	145	1352	**0.0244**	0.3182	**<.0001**	**<.0001**
2. Feedback												
a. Knowledge of performance (unless corrective)	108	146	6.1	13	0	0	0	0	**<.0001**	**<.0001**	**<.0001**	**<.0001**
b. KP and positive social stimuli	84.7	129	3.7	10	0	0	0	0	**<.0001**	**<.0001**	**<.0001**	**<.0001**
c. KP and negative social stimuli	0	0	0.1	< 1	0	0	0	0	0.4801	0.4801	0.5548	0.5548
d. Corrective KP (cKP)	1.0	6.0	0.7	1	0	0	0	0	**0.0119**	0.1030	0.6804	0.2169
e. cKP and positive social stimuli	0	0	0	0	0	0	0	0				
f. cKP and negative social stimuli	0	0	0	0	0	0	0	0				
g. Knowledge of result	0	0	82.5	219	0	0	35.9	313	**0.0001**	**0.0036**	**<.0001**	**<.0001**
h. KR and positive social stimuli	0.2	< 1	42.9	108	0	0	4.3	34	**0.0001**	**0.0044**	**0.0001**	**<.0001**
i. KR and negative social stimuli	0	0	0	0	0	0	0	0				
3. Motivational interactions												
a. Other than KP or KR	0.2	< 1	3.4	8	0.9	7	2.0	13	0.4074	**0.0033**	**0.0019**	**0.0011**
4. Bond												
a. Showing interest in person	8.8	43	35	75	17	118	44.5	289	**0.0005**	**<.0001**	**<.0001**	**<.0001**
b. Personal aspects (therapist)	0	0	0.8	11	0	0	0	0	**0.0435**	0.0618	0.0889	0.1159
c. Responsivity	9.0	42	44.9	157	0	0	0	0	**0.0011**	**0.0003**	**0.0446**	**0.0443**
d. Conflict solving	0.3	5.5	0.3	4	0	0	0	0	0.0546	0.1037	1.0000	0.8524
5. Other types of interaction	0.5	4.3	1.6	9	0	0	0	0	**0.0007**	**0.0068**	0.0882	0.3691
6. Presence (concentration) and engagement (treating person) (0–10)	9.0		8.8		5.0		5.0		**<.0001**		0.5950	
7. Focussed attention and engagement (patient) (0–10)	9.5		8.3		8.3		8.8		0.6730		0.3700	
Length of the therapeutic session (minutes)	71		81		77		107		**0.0006**		**0.0001**	

ABT, arm basis training; AAT, arm ability training; Fr, frequency of occurrence of the therapeutic interaction within the session (count) rounded to one decimal; Ti, time used for the therapeutic interaction within the session (in seconds) rounded to full seconds; KP, knowledge of performance; KR, knowledge of result; *p*-values correspond to F statistics based on type III sums of squares of ANOVA. Bold values denote *p*-values < .05.

Overall, information provided by the humanoid robot included treatment-goal-oriented communication, training specifications, and instructions. Treatment-goal-oriented therapeutic interaction was characterized by a few more extended explanatory communication episodes. By far, the most frequently observed therapeutic interaction had been brief instructions, both for the ABT and AAT, while being both more frequent and shorter for the ABT compared to AAT. Training specifications (how the training is structured and how it might work) had been observed with the AAT only as a single longer explanation period per training session.

Feedback had only been observed with AAT and was provided as KR, mostly presented in a neutral manner and at times combined with positive social stimuli.

Bond-related interactions were also not infrequently documented, fell in the category “showing interest in the person treated” (e.g., asking the patient whether she or he is ready to continue), and were observed more frequently during AAT sessions.

The therapeutic interaction by a supervising therapist was comparatively infrequent, mostly observed in AAT sessions, mainly as additional instructions and some bond-related activity (in categories showing interest in the other person and responsivity).

Interaction by a helper for physical assistance (ABT only) was again much less frequent than interaction episodes by the humanoid robot and included mainly instructions, some feedback as KP, and not infrequently showing interest in the other person.

#### 3.2.2 Humanoid robot interaction—Its changes across sessions

The pattern of therapeutic interaction by the humanoid robot and its changes across sessions are presented in Table 3.

On the first day, one characteristic of the humanoid robot’s therapeutic interaction was a considerable degree of information provision. As therapy progressed, patients became more knowledgeable about the training and were given less information provision (frequency and time allocated), while information was still offered by the humanoid robot as an option. As a consequence, more time was available for executing training tasks and led to more instructions.

For AAT, a small shift from “neutral” knowledge of the result feedback to the feedback associated with positive social stimuli (given with greater improvements within or across sessions) was observed from day 1 to day 9 of humanoid robot-led therapy indicating even better progress on day 9.

Presence and engagement rating for patients did not change from day 1 to day 9 indicating a high degree of focused attention and engagement performing the training tasks both from the beginning and being persistent over the course of daily therapy with a humanoid robot.

#### 3.2.3 Pattern of the therapeutic interaction by the humanoid robot compared to human therapists providing the same type of treatment

Generally speaking, the pattern of the therapeutic interaction by the humanoid robot and human therapists providing the same type of treatment was fairly comparable with regard to the provision of information, feedback, and bond-related interaction ( Table 4).

A closer look, nevertheless, documented differences for the therapeutic interaction by the humanoid robot and human therapists. A slightly more treatment-goal-related interaction (ABT) by the humanoid robot agent was observed, that is, comments regarding the training with reference to individual baseline scores and training goals. With humanoid robot therapy, less-frequent (repeated) instructions for individual training movements (ABT), no knowledge of performance (KP) feedback (ABT), and less knowledge of result (KR) feedback (AAT) (human therapist spontaneously provided not only summary KR but also additional immediate KR) were given. The robot showed more episodes of interest in the other person (e.g., asked “are you ready?“) while a human therapist presumably perceived such information more frequently without having to ask. The humanoid robot lacked responsivity to spontaneous cues by patients (a fact that did, however, not lead to a necessity to solve conflicts). Overall, the robot was rated as less “engaged and present” by an independent offline rater compared to a human therapist, and it “did its job well,” but was perceived and rated not to act as close/attentive to patients’ behavior and needs as human therapists did. Patients training with a humanoid robot, nevertheless, showed similarly focused attention and engagement compared to patients having therapy sessions with a human therapist. Therapeutic sessions were somewhat longer with a robot (as intended) resulting in substantially long therapeutic sessions.

## 4 Discussion

Stroke survivors who participated in this research all had a need for arm rehabilitation, but were otherwise diverse with regard to their characteristics including gender, age, type of and time post-stroke, degree of overall disability (mild to moderate), and emotional distress (comparing Table 1). Collectively, the study population and its sub-groups receiving human- or humanoid robot-led therapy represented the typical range of characteristics that therapists encounter when providing stroke rehabilitation. Also, the sub-groups of stroke survivors receiving therapy by a human therapist or the robot-led therapy were largely comparable. If anything, the participants in the robot group had slightly more pronounced neuro-disability on average and, hence, might have generated a somewhat more challenging therapeutic situation (comparing Table 1).

In addition, the therapeutic situation (outpatient or inpatient scenario) was comparable to other regular rehabilitation treatments offered in medical centers.

Given both the study population characteristics and the therapeutic situation, the study context resembled regular treatment scenarios for stroke rehabilitation well and, therefore, promotes the ecological validity of the data generated; that is, the observations made can be considered relevant for routine clinical practice.

The pattern of the therapeutic interaction as provided by the humanoid robot included episodes of provision of information, feedback, and bond-related interaction (comparing Table 2) and while sharing similarities varied, nevertheless, across therapies (i.e., ABT and AAT, respectively; for statistical analyses, we compare Tables 2–4).

The humanoid robot addressed the issue of an individual treatment goal, explained the mechanism of action of therapy extensively (AAT), provided frequent brief instructions, intermittently feedback as knowledge of results (AAT), and showed interest in the treated person’s situation (e.g., whether a patient was ready to continue with the next exercise).

The helper who provided physical assistance with weight bearing and movement of the more severely affected arm when needed (ABT only) and who was not a therapist, but based her or his activities on the system’s instructions and prompts, added spontaneously further instructions (e.g., “you need to …”) and communication episodes that showed interest in the other person (e.g., “are you ready?”). As a consequence, the supervising therapist contributed very little additional therapeutic interactions in ABT sessions while similarly adding spontaneously further instructions and communication episodes that showed interest in the other person (e.g., “are you ready?”) during AAT sessions (without a helper being present). Taken together, these observations indicate that the humanoid robot covered the therapeutic interaction by and large sufficiently. During these humanoid robot-led therapy sessions, the human person being closest to the patient receiving therapy (i.e., the helper with ABT and supervising therapist with AAT) still occasionally spontaneously stepped in, mainly providing additional instructions and addressing personal context issues (e.g., being ready to continue). The data cannot tell whether such interaction was mandatory for the session’s success. At any rate, it seemed not necessary to solve any conflict of risk of discontinuation of the therapeutic sessions as this would have fallen into the corresponding interaction category (i.e., conflict solving) that was rarely ever observed.

Over time (comparing day 9 to day 1 of training with the humanoid robot), the stroke survivors needed less general information and, hence, had more time for executing training tasks. The humanoid robot adapted its behavior, provided less information (while still offering it), and executed more instructions accordingly (comparing Table 3). The measures for focused attention and engagement were at a high level (on average, between 8 and 9 on a scale from 0 to 10) and constant across sessions indicating that the training (ABT and AAT) and working with the humanoid robot intensively over 9 days were suitable to both induce and stabilize a high degree of focused attention and engagement among the treated stroke survivors. The observation is considered important since “neural repair therapy” meant to improve brain functions by specific and intensive training can only be successful if such attitudes can be achieved and maintained during training.

Finally, the research intended to compare the observed therapeutic interaction as provided by the humanoid robot in therapy session situations to the therapeutic interaction provided by human therapists providing the same type of therapy in a conventional 1:1 therapeutic setting. Here, the overall picture was that the humanoid robot’s therapeutic interaction resembled the therapeutic interaction by human therapists well, and even differences between therapies were well matched. This is reassuring since the therapeutic system E-BRAiN was developed to lead through (daily) therapy sessions in an autonomous way with all communication and therapeutic interaction necessary.

Differences documented between the humanoid robot’s and human therapists’ therapeutic interaction are, nevertheless, worthwhile noting. It is considered a strength of the therapeutic system E-BRAiN that it links individual treatment goals with the training prescribed even slightly more frequently than human therapists do. Other differences are, however, related to technical limitations of the current technology used and algorithms implemented. So far, the system cannot sense limb movements or muscle innervation and, hence, cannot provide KP during ABT as humans can, based on their visual and tactile perception of innervation and movement attempts by patients, and similarly is limited to provide additional instructions based on partial completion of movements. The system also cannot recognize and interpret spontaneous verbal and non-verbal communication cues provided by patients and cannot be responsive to them. Indeed, related research indicated that stroke survivors consider it a relevant disadvantage that currently available socially interactive humanoid robot systems do not possess human abilities, such as the ability to hold a conversation and to express or understand emotions (Dembovski et al., 2022). Future further development of the system might help to overcome some of these limitations.

It is, however, of importance to note in this context that focused attention and engagement by patients during the training sessions observed were high and comparable for both humanoid robot- and human therapist-led therapy sessions, not only when the series of training sessions commenced (day 1) but also after nine daily sessions (day 9). Thus, any differences in the therapeutic interaction observed did not translate in a different behavioral attitude of patients, and even somewhat longer therapeutic sessions could be realized.

The digital therapy system E-BRAiN uses a socially interactive humanoid robot as technology and established AI that provides (A) professional therapeutic training knowledge for both arm rehabilitation and neglect therapy based on types of therapy with evidence to support their effectiveness for recovery post-stroke (in this research, demonstrated for ABT and AAT), (B) effectively leads through (daily) therapy sessions in an autonomous way with all communication and therapeutic interaction necessary, and (C) individualizes all activities based on individual data (e.g., clinical characteristics, results of assessment, and therapeutic goal).

The systems that had been developed so far equally demonstrated that socially interactive humanoid robots can be used for arm rehabilitation after stroke in a clinically meaningful and acceptable way (Koren et al., 2022).

Here, we add to our knowledge that such a system can be set up in a way that not only generates a sequence of tasks to be practiced but also provides the means for a series of largely autonomous humanoid robot-led therapeutic sessions with all types of therapeutic interaction necessary. Furthermore, the system E-BRAiN integrates personalized information that adapts the system’s behavior to individual needs, ongoing training behavior, and progress. This is even true for different forms of therapies that can be prescribed as needed based on individual clinical circumstances. All these refined aspects of AI integration led to the overall comparability of therapeutic interaction during humanoid robot-led sessions using E-BRAiN with interaction observed during conventional human therapist-led therapeutic sessions providing the same type of therapy.

The research, thus, provides evidence that AI using humanoid robot technology together with algorithms to implement complex rehabilitation therapy assistance can achieve scenarios that resemble human–patient interactions, comprehensively represent the work flow of therapeutic sessions for training-based therapies with strong evidence to support their clinical effectiveness, and might, therefore indicate a way to establish more specific and intensive “neural repair” therapy.

Given the increasing global societal need to combat neuro-disabilities, such solutions could play a pivotal role once established, when proven to be acceptable to people with neuro-disabilities in need for rehabilitation and to be clinically safe and (cost-)effective.

Perceived from a broader perspective, robot technology that may be used for rehabilitation purposes might provide either specific therapeutic interaction (as investigated here), register training behavior by sensor technology, and/or provide physical assistance as needed. Ideally, rehabilitation technology could be equipped with some or all of these characteristics, depending on specific use cases.

Indeed, mechanical robot technology providing physical assistance as needed for repetitive practice has effectively been introduced in neurorehabilitation and helps to enhance intensive repetitive practice schedules, especially among people with severe paresis (e.g., after stroke) (Mehrholz et al., 2018). First applications for human care also demonstrate that humanoid aspects can be integrated into applications that provide physical assistance, for example, for daily care or physical therapy practice (Mukai et al., 2010; Jevtić et al., 2019; Miyake et al., 2022). With regard to therapy they do, however, lack comprehensive social interaction that supports a rehabilitation technology to be used without close supervision by human therapists.

In the research reported, a comprehensive social therapeutic interaction by a humanoid robot implemented in and used with a therapeutic system has been characterized and shown to be largely comparable to the human therapeutic interaction when providing the same types of therapy. While the system can also record training progress for some aspects (e.g., the time used for AAT tasks to be completed), but so far not for others (e.g., selective motion for the various joints as practiced during the ABT), it cannot provide physical assistance, an aspect that is compensated for by a human helper in the context of the ABT (while not needed for the AAT, a training for people with mild arm paresis).

For the future, it is well conceivable that systems could be developed that comprehensively integrate specific therapeutic interaction (as investigated here), register training behavior by sensor technology more comprehensively (e.g., motion tracking), and/or provide physical assistance as needed.

## Data Availability

The datasets presented in this article are not readily available because the data presented may only be used by personal and for purposes that had been agreed upon in advance in writing by participants. Requests to access the datasets should be directed to thomas.platz@uni-greifswald.de.
